# Prognostic impact of platelet-to-lymphocyte ratio on diffuse large B-cell lymphoma: a meta-analysis

**DOI:** 10.1186/s12935-019-0962-3

**Published:** 2019-09-24

**Authors:** Ying Chen, Zongxin Zhang, Qiu Fang, Huiqin Jian

**Affiliations:** 1 Clinical Laboratory, Huzhou Central Hospital, Huzhou, 313000 Zhejiang China; 2 Affiliated Central Hospital of Huzhou University, Huzhou Central Hospital, Huzhou, 313000 Zhejiang China; 3 Department of Hematology, Huzhou Central Hospital, Huzhou, 313000 Zhejiang China

**Keywords:** Meta-analysis, PLR, Diffuse large B-cell lymphoma, Prognosis, Biomarker

## Abstract

**Background:**

Recently, some studies reported the prognostic value of platelet-to-lymphocyte ratio (PLR) in patients with diffuse large B-cell lymphoma (DLBCL), however, the results varied from different studies. Therefore, we performed a meta-analysis to explore the prognostic value of PLR in DLBCL.

**Methods:**

A comprehensive literature retrieval was conducted by using PubMed, Embase, Web of Science, the Cochrane Library, the China National Knowledge Infrastructure (CNKI), and Wanfang. Pooled hazard ratio (HR) and 95% confidence interval (CI) were used to evaluate the association of PLR and overall survival (OS) and progression-free survival (PFS). Odd ratios (ORs) and 95% CIs for clinicopathological characteristics were statistically analyzed.

**Results:**

Eight studies with 1931 patients were included for meta-analysis. The pooled analysis indicated that elevated PLR was significantly associated with poor OS (HR = 1.73, 95% CI 1.29–2.31, p < 0.001), but not PFS (HR = 0.85, 95% CI 0.57–1.27, p = 0.438). Furthermore, elevated PLR was significantly associated with presentation of B symptoms (OR = 2.27, 95% CI 1.29–3.98, p = 0.004), elevated lactate dehydrogenase (LDH) (OR = 2.76, 95% CI 2.05–3.72, p < 0.001), higher tumor stage (OR = 2.22, 95% CI 1.66–2.98, p < 0.001), and Eastern Cooperative Oncology Group (ECOG) performance status (PS) ≥ 2 (OR = 1.71, 95% CI 1.09–2.69, p = 0.019). However, elevated PLR was not significantly correlated with gender, age or cell of origin.

**Conclusion:**

This meta-analysis revealed that PLR may be an effective and noninvasive biomarker for poor prognosis and aggressive disease characteristics for patients with DLBCL.

## Background

Diffuse large B-cell lymphoma (DLBCL) is the most frequent type of non-Hodgkin lymphoma, accounting for approximately 30–40% of all malignant lymphomas worldwide [1, 2]. DLBCL presents heterogeneous and aggressive status with different biological and clinical features [3]. Since rituximab (R)-cyclophosphamide, doxorubicin, vincristine, and prednisone (R-CHOP) regimen became the standard treatment over the past decade, over 60% of DLBCL patients are curable, whereas approximately 30% of patients experience primary refractory or relapsed disease [4]. Prognostic biomarkers are important for survival prediction and therapeutic strategies selection. International prognostic index (IPI) is widely used for prognosis of DLBCL, however, the prognostic efficiency still needs to be improved [5]. Therefore, search of cost-effective and easily available prognostic markers is of high importance for DLBCL treatment.

Inflammatory responses are involved in different steps of cancer development [6]. Inflammation activity plays an important role in prognostication. The indexes derived from hematological parameters are investigated for prognosis of cancer patients in recent years. Platelet-to-lymphocyte ratio (PLR) is calculated as platelet counts divided by lymphocyte counts. PLR was shown to be a significant prognostic marker in various solid tumors including esophageal cancer [7], head and neck squamous cell carcinoma [8], ovarian cancer [9], and breast cancer [10]. Previous retrospective studies also explored the prognostic effect of PLR in DLBCL, whereas the results were inconsistent even contrary [11–16]. Therefore, it is necessary to conduct a meta-analysis to comprehensively evaluate the prognostic and clinicopathological role of PLR in DLBCL patients.

## Materials and methods

### Search strategy

We performed the present meta-analysis based on the Preferred Reporting Items for Systematic Reviews and Meta-Analyses (PRISMA) Statement [17]. The databases including PubMed, Web of Science, Cochrane Library, Embase, CNKI (Chinese), and Wanfang were searched for relevant studies. The search strategies included the combination of MeSH terms and free-text terms: “platelet-to-lymphocyte ratio,” “platelet–lymphocyte ratio,” “PLR,” and “diffuse large B-cell lymphoma or DLBCL”. The last search was up to May, 2019. In addition, the references list of relevant researches was examined to identify relevant studies. Ethical approval was not required for this study because all data were from previous published studies.

### Selection criteria

The inclusion criteria were following: (1) patients with DLBCL were diagnosed histologically; (2) studies reported the prognostic role of PLR on overall survival (OS) or/and progression-free survival (PFS) or provided sufficient data for calculation [18]; (3) a cut-off value of PLR was identified; (4) studies were published in English or Chinese; (5) the laboratory parameters including the blood counts were assessed prior to the start of first chemotherapy cycle. Studies did not meet the inclusion criteria were removed.

### Data extraction and quality evaluation

Two experienced investigators (Y.C. and Z.Z.) independently extracted the data. Any discrepancies were resolved by discussion with the third investigator (H.J.). The main characteristics of eligible studies were: name of first author, year of publication, country, sample size, patients’ age, gender, treatment regimen, stage, cut-off value of PLR, study period, and survival outcomes. The quality assessment of included studies were performed according to Newcastle–Ottawa Scale (NOS) [19]. The NOS consists of three parts: selection (0–4 points), comparability (0–2 points), and outcome (0–3 points). The studies of highest quality scored 9 points and studies ≥ 6 points indicated a high quality.

### Statistical analysis

All statistical analyses were performed by using Stata version 12 (STATA, College Station, TX). Pooled hazard ratio (HR) and 95% confidence interval (CI) were used to evaluate the association of PLR and OS and PFS. HR and 95% CI were extracted from included studies if reported, or were calculated from Kaplan–Meier curves by Tierney’s method [18]. The heterogeneity across studies was assessed by Cochran’s Q test and I-squared test [20, 21]. A random-effect model was employed when the heterogeneity was significant (p < 0.10 or *I*^2^ > 50%), otherwise, a fixed model was applied. Odd ratios (ORs) and 95% CIs for clinicopathological characteristics were statistically analyzed. Sensitivity was performed by sequential omitting of each study to test the credibility of the results. The potential publication bias was examined by Begg’s funnel plot and Egger’s test. A two-tailed p-value < 0.05 was defined as statistically significant.

## Results

### Search results

**Fig. 1:**
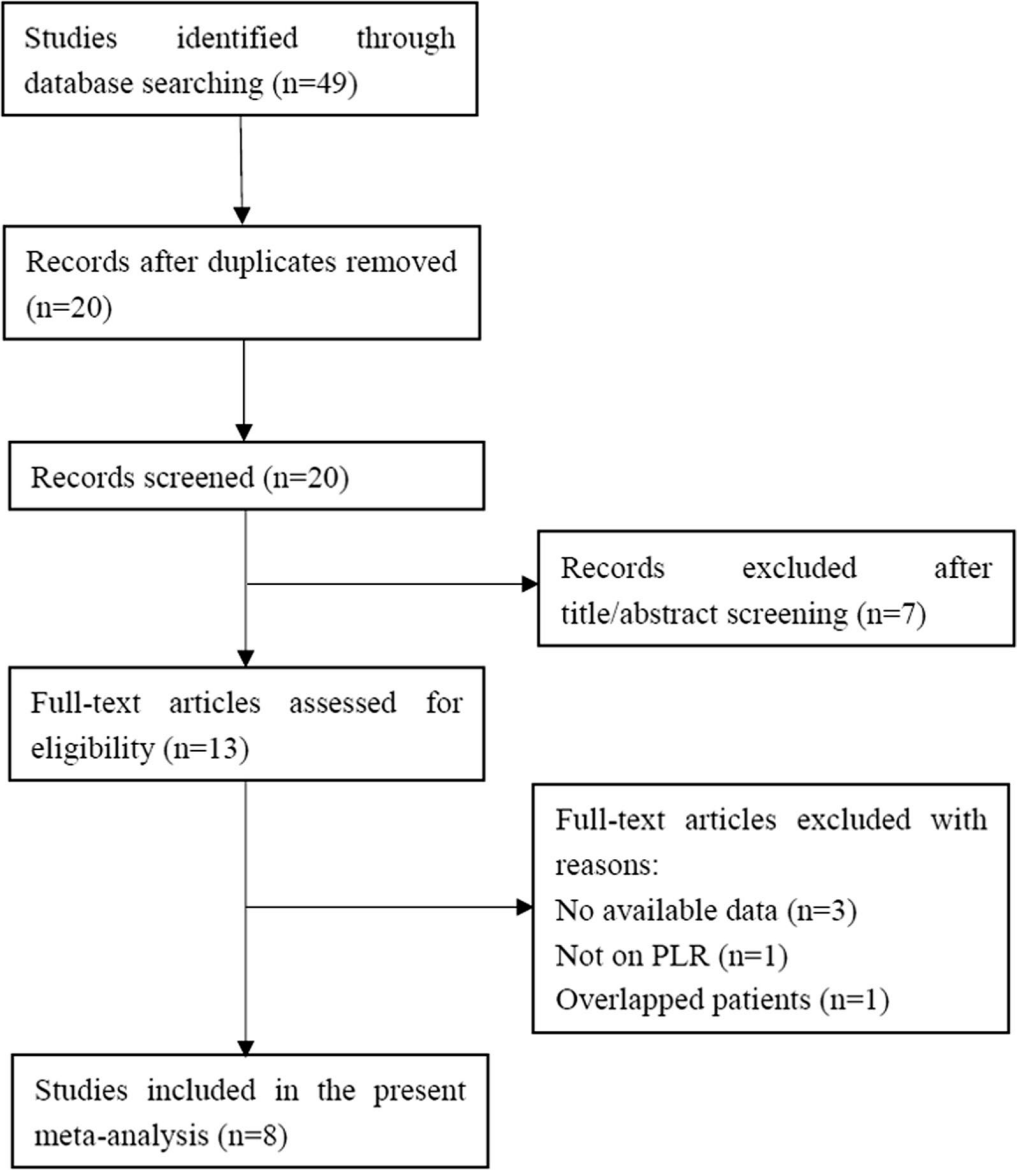
Flow diagram of study selection

A total of 49 studies were identified after initial literature search. After duplicate records were removed, 20 studies remained. Seven records were excluded by title/abstract screening and 13 studies were left for full-text evaluation. Five full-text articles were removed for reasons: no available data, not on PLR, and overlapped patients. At last, eight studies [11–16, 22, 23] were included in the current meta-analysis. The detailed process of literature retrieval was depicted in Fig. 1.

### Study characteristics

The eight studies were published from 2015 to 2019. Six studies were conducted in China [12, 14–16, 22, 23], one in Austria [11] and one in Croatia [13]. Five studies were published in English [11, 13, 14, 16, 22] and three in Chinese [12, 15, 23]. The total sample size was 1931, ranging from 59 to 515. The cut-off values of PLR ranged from 143 to 435. Five studies [11–13, 16, 22] employed R-CHOP regimen and three studies [14, 15, 23] used R-CHOP/CHOP regimen. All eight studies [11–16, 22, 23] reported the prognostic value of PLR on OS and seven studies [12–16, 22, 23] showed the association of PLR and PFS. All included studies had a NOS score ≥ 6. The baseline characteristics of included studies were summarized in Table 1.

**Table 1:** Basic characteristics of the studies enrolled

Author	Year	Country	Ethnicity	Sample size	Sex (M/F)	Age (year) Median (range)	Stage	Treatment	Cut-off	Duration	NOS score	Survival outcome
Melchardt	2015	Austria	Caucasian	515	270/245	65 (20–92)	I–IV	R-CHOP	435	2004–2014	8	OS
Ni	2016	China	Asian	59	36/23	54 (14–75)	I–IV	R-CHOP	270	2009–2015	8	OS, PFS
Perisa	2016	Croatia	Caucasian	103	37/66	63 (22–87)	I–IV	R-CHOP	162	2006–2015	7	OS, PFS
Hao	2017	China	Asian	252	165/87	49 (16–82)	I–-IV	R-CHOP/CHOP	150	2003–2014	8	OS, PFS
Han	2018	China	Asian	361	203/158	55 (12–91)	III–IV	R-CHOP/CHOP	300	2006–2012	6	OS, PFS
Wang	2018	China	Asian	182	96/86	59 (18–80)	I–IV	R-CHOP	150	2005–2016	7	OS, PFS
Zhao	2018	China	Asian	309	186/123	58 (16–90)	I–IV	R-CHOP	170	2009–2015	7	OS, PFS
Lin	2019	China	Asian	150	96/54	56 (15–94)	I–IV	R-CHOP/CHOP	143	2013–2017	6	OS, PFS

R-CHOP: rituximab, cyclophosphamide, doxorubicin, vincristine, and prednisone; CHOP: cyclophosphamide, doxorubicin, vincristine, and prednisone; OS: overall survival; PFS: progression-free survival

### PLR and OS

**Fig. 2:**
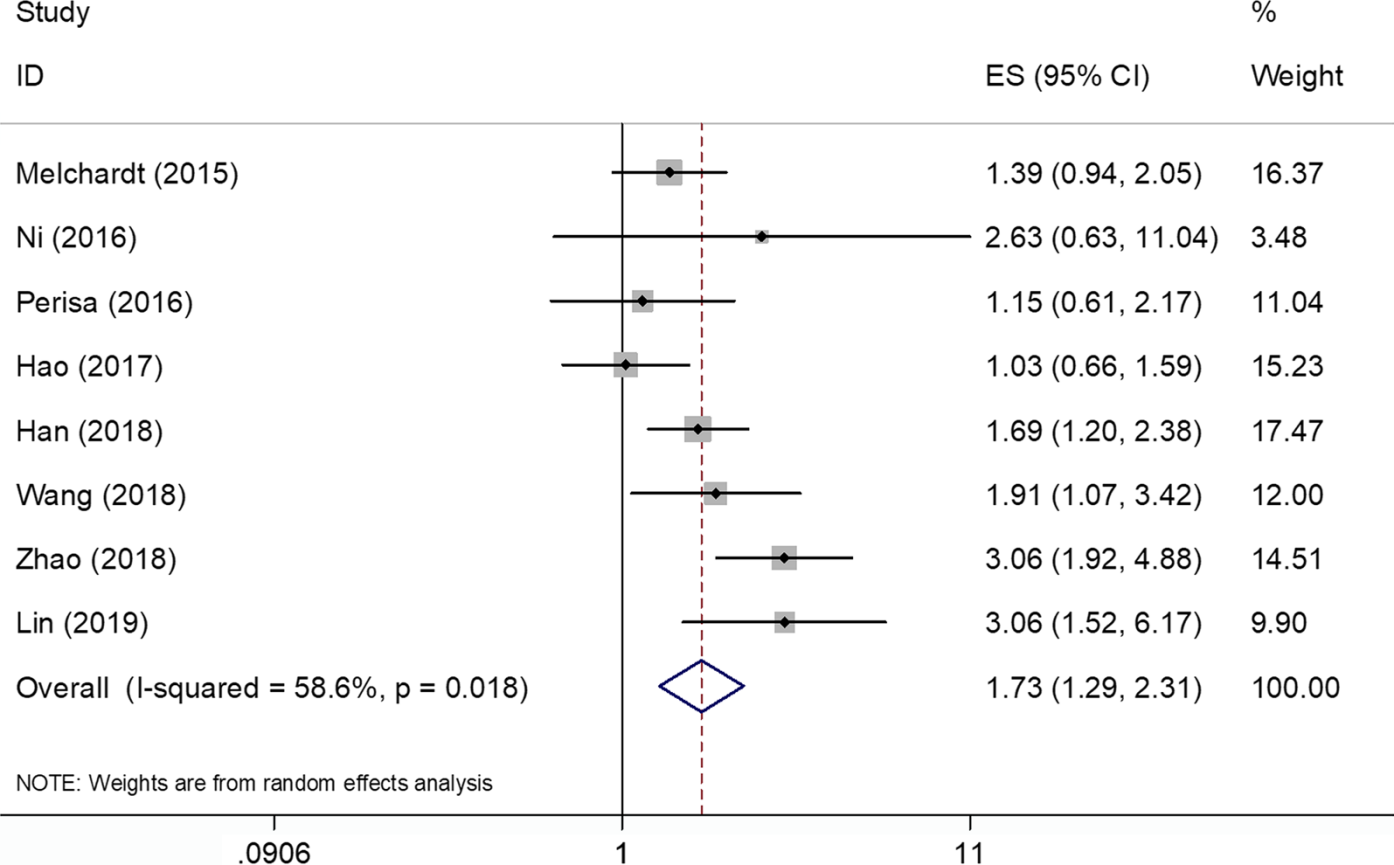
Forest plots of studies evaluating the relationship between PLR and overall survival (OS)

All eight studies [11–16, 22, 23] with 1931 patients showed the correlation between PLR and OS. The random-effect model was used due to significant heterogeneity (Ph = 0.018, *I*^2^ = 58.6%; Table 2, Fig. 2). The pooled analysis showed that a high PLR was significantly correlated to worse OS (HR = 1.73, 95% CI 1.29–2.31, p < 0.001) (Fig. 2, Table 2). To yield a further investigation, the subgroup analyses were conducted. The pooled data indicated that PLR was still a significant prognostic marker in Asian patients (HR = 1.95, 95% CI 1.34–2.84, p < 0.001), in studies with sample size ≤ 200 (HR = 1.88, 95% CI 1.32–2.67, p < 0.001), in studies with sample size > 200 (HR = 1.63, 95% CI 1.09–2.45, p = 0.018), and cut-off > 150 (HR = 1.76, 95% CI 1.25–2.49, p = 0.001) (Table 2). However, the prognostic value was non-significant for PLR in Caucasian patients (HR = 1.32, 95% CI 0.95–1.84, p = 0.103) or cut-off value ≤ 150 (HR = 1.74, 95% CI 0.92–3.28, p = 0.088) (Table 2).

**Table 2:** Results of subgroup meta-analysis

Characteristics	No. of studies	No. of patients	Effects model	HR (95% CI)	p	Heterogeneity	
						*I*^2^ (%)	Ph
OS							
All	8	1931	Random	1.73 (1.29–2.31)	< 0.001	58.6	0.018
Ethnicity							
Caucasian	2	618	Fixed	1.32 (0.95–1.84)	0.103	0	0.624
Asian	6	1313	Random	1.95 (1.34–2.84)	< 0.001	64.2	0.016
Sample size							
≤ 200	4	494	Fixed	1.88 (1.32–2.67)	< 0.001	31.7	0.222
> 200	4	1437	Random	1.63 (1.09–2.45)	0.018	75	0.007
Cut-off value							
≤ 150	3	584	Random	1.74 (0.92–3.28)	0.088	73.5	0.023
> 150	5	1347	Random	1.76 (1.25–2.49)	0.001	55	0.064
PFS							
All	7	1416	Random	0.85 (0.57–1.27)	0.438	77.8	< 0.001
Ethnicity							
Caucasian	1	103	–	0.57 (0.3–1.09)	0.091	–	–
Asian	6	1313	Random	0.91 (0.58–1.41)	0.66	80	< 0.001
Sample size							
≤ 200	4	494	Random	0.67 (0.34–1.34)	0.26	74.7	0.008
> 200	3	922	Random	1.08 (0.73–1.62)	0.69	72.8	0.025
Cut-off value							
≤ 150	3	584	Random	0.62 (0.32–1.21)	0.161	80.1	0.007
> 150	4	832	Random	1.12 (0.76–1.65)	0.58	54.7	0.085

### PLR and PFS

**Fig. 3:**
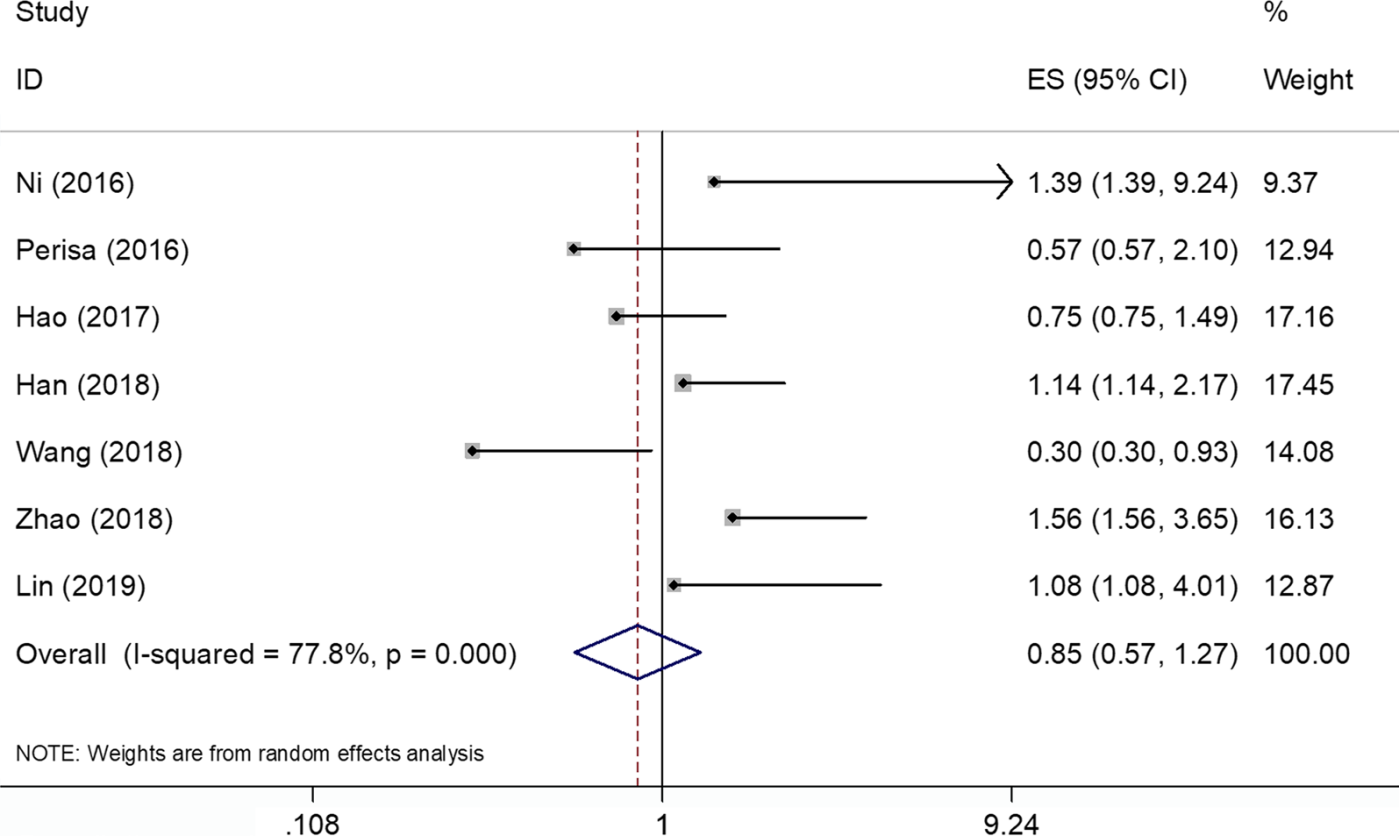
Forest plots of studies evaluating the relationship between PLR and progression-free survival (PFS)

Seven studies [12–16, 22, 23] including 1416 patients reported the association of PLR and PFS. Because of significant heterogeneity (Ph < 0.001, *I*^2^ = 77.8%; Table 2, Fig. 3), a random-effect model was applied. The combined results were HR = 0.85, 95% CI 0.57–1.27, p = 0.438 (Table 2, Fig. 3), indicating that PLR was not a prognostic factor for PFS. Subgroup analysis demonstrated that PLR was not correlated to PFS irrespective of ethnicity, sample size, or cut-off value of PLR.

### PLR and clinicopathological characteristics

The correlation of PLR and several clinicopathological characteristics were analyzed. The clinicopathological features included: B symptoms (yes vs no), lactate dehydrogenase (LDH) level (> normal vs < normal), tumor stage (III–IV vs I–II), gender (male vs female), age (≥ 60 vs < 60), Eastern Cooperative Oncology Group (ECOG) performance status (PS) (≥ 2 vs < 2), and cell of origin [germinal center B cell (GCB) vs non-GCB]. As shown in Table 3, the pooled analysis showed that PLR was significantly associated with presentation of B symptoms (OR = 2.27, 95% CI 1.29–3.98, p = 0.004), elevated LDH (OR = 2.76, 95% CI 2.05–3.72, p < 0.001), higher tumor stage (OR = 2.22, 95% CI 1.66–2.98, p < 0.001), and ECOG PS ≥ 2 (OR = 1.71, 95% CI 1.09–2.69, p = 0.019). However, PLR was not significantly correlated with gender, age or cell of origin.

**Table 3:** Correlation of PLR and clinical factors

Characteristics	No. of studies	No. of patients	Effects model	OR (95% CI)	p	Heterogeneity	
						*I*^2^ (%)	Ph
B symptoms (yes vs no)	5	803	Random	2.27 (1.29–3.98)	0.004	60.2	0.039
LDH (> normal vs < normal)	5	803	Fixed	2.76 (2.05–3.72)	< 0.001	0	0.703
Stage (III–IV vs I–II)	5	803	Fixed	2.22 (1.66–2.98)	< 0.001	21.1	0.28
Gender (male vs female)	5	803	Fixed	0.9 (0.67–1.19)	0.447	3.8	0.385
Age (≥ 60 vs < 60)	5	803	Fixed	0.91 (0.69–1.21)	0.527	0	0.875
ECOG PS (≥ 2 vs < 2)	4	494	Fixed	1.71 (1.09–2.69)	0.019	0	0.745
Cell of origin (GCB vs non-GCB)	2	491	Fixed	0.94 (0.65–1.38)	0.765	0	0.740

LDH: lactate dehydrogenase; ECOG PS: Eastern Cooperative Oncology Group performance status; GCB: germinal center B cell

### Sensitivity analysis

**Fig. 4:**
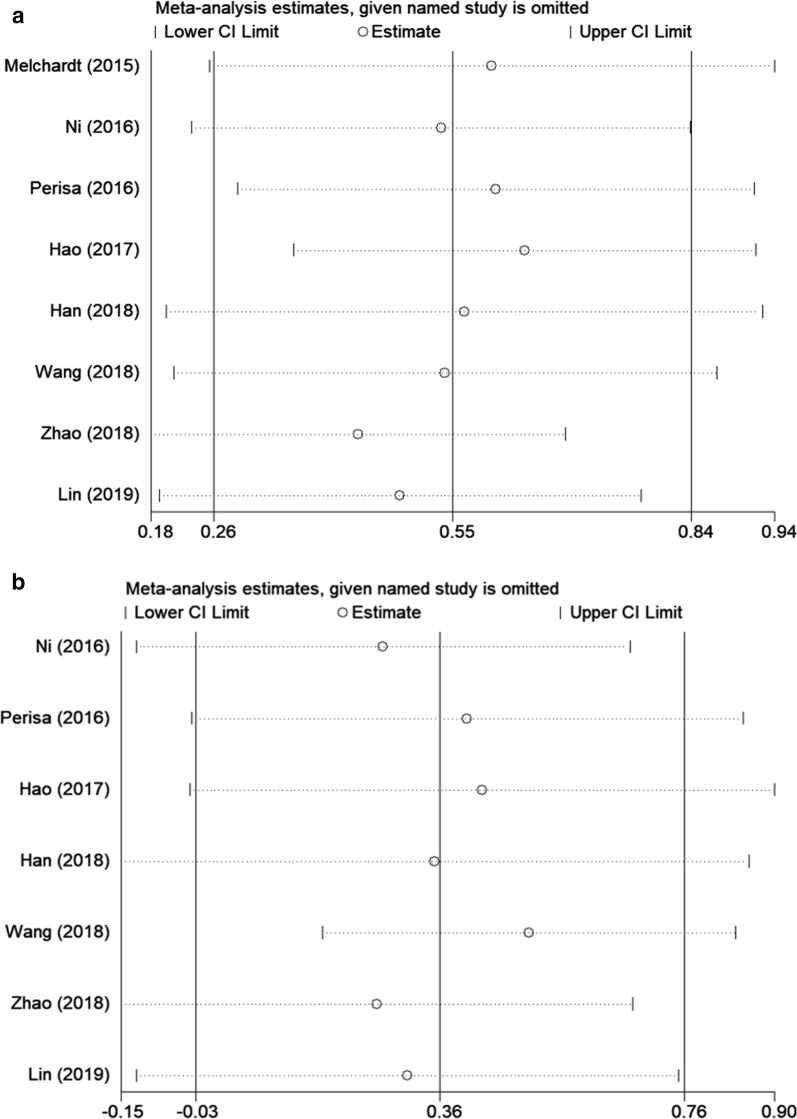
Sensitivity analysis of the influence of each individual study on the pooled hazard ratios (HRs) for the relationship between PLR and **a** OS and **b** PFS

To evaluate the influence of each single study of the pooled results, sensitivity analysis was carried out. As shown in Fig. 4, the pooled HRs were not significantly altered by any individual study, which indicated the stability of the results.

### Publication bias

**Fig. 5:**
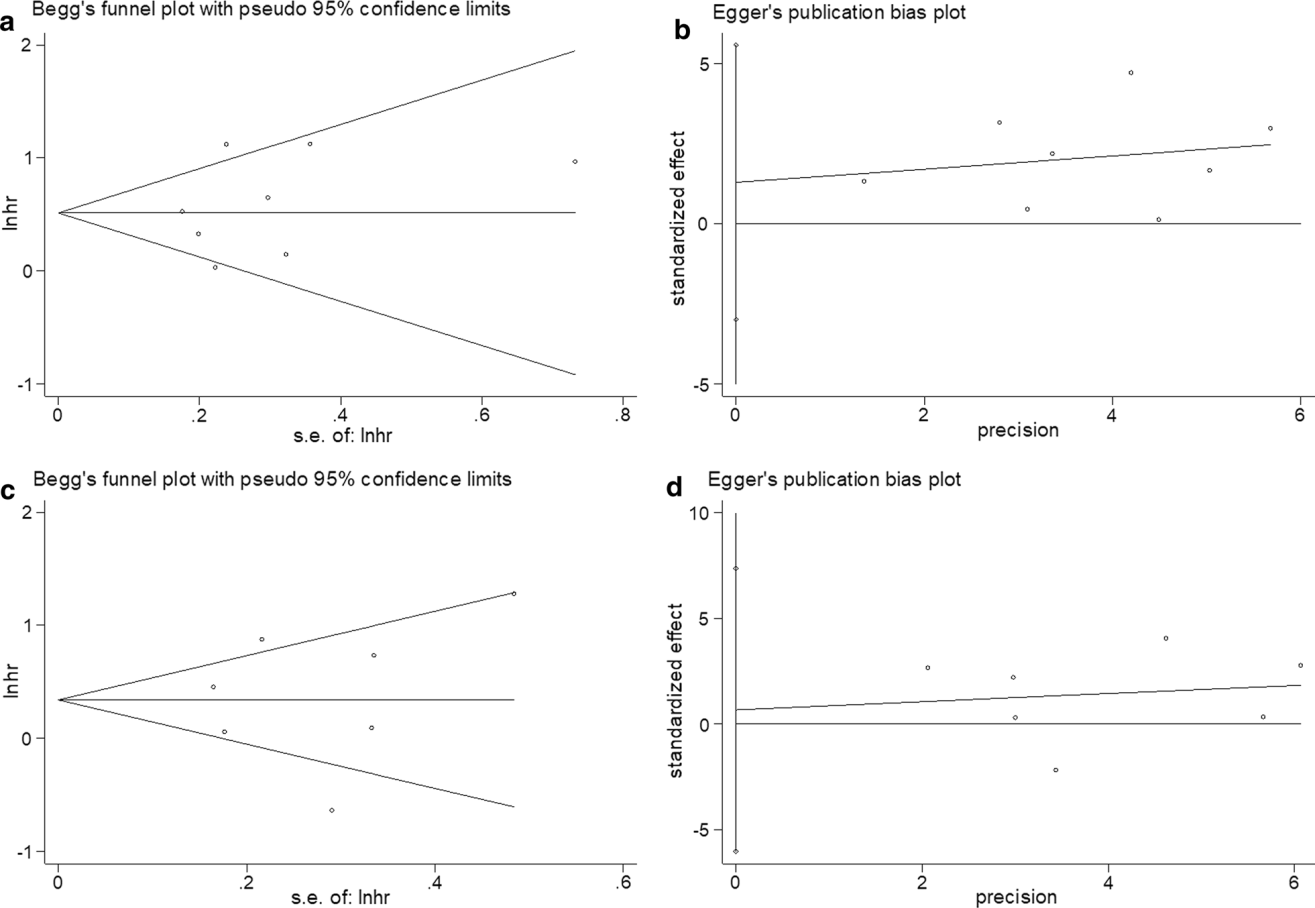
Publication bias: **a** Begg’s test for OS, **b** Egger’s test for OS, **c** Begg’s test for PFS, and **d** Egger’s test for PFS

The results of Begg’s funnel plot (OS, p = 0.536; PFS, p = 0.548) and Egger’s test (OS, p = 0.489; PFS, p = 0.808) indicated no publication bias in the present meta-analysis (Fig. 5).

## Discussion

Inflammation plays a pivotal role in tumor progression [24]. PLR was widely investigated for prognosis of DLBCL patients with distinct results. Previous studies reported the prognostic value of PLR in DLBCL patients [11–16, 22, 23], whereas the results were inconsistent. For example, some studies [22, 23] demonstrated that PLR was a significant prognostic factor for DLBCL patients, whereas other studies failed to find the prognostic value of PLR [11, 13, 14]. As meta-analysis can aggregate data from a series of studies and make quantitative analysis, therefore, the results of meta-analysis are objective and credible.

In the present meta-analysis, we aggregated data from eight studies with 1931 patients to shed light on this issue. The results showed that a high PLR was a significant prognostic marker for poorer OS (HR = 1.73, 95% CI 1.29–2.31, p < 0.001). Furthermore, the prognostic efficiency enhanced for Asian patients (HR = 1.95, 95% CI 1.34–2.84, p < 0.001) and with a cut-off value > 150 (HR = 1.76, 95% CI 1.25–2.49, p = 0.001). However, PLR was not associated with PFS in DLBCL, regardless of ethnicity, sample size, or cut-off value of PLR. We also found that PLR was significantly correlated to presentation of B symptoms, elevated LDH, higher tumor stage, and ECOG PS ≥ 2. The results suggested that PLR was positively connected with clinical features reflecting high aggressiveness of the disease. Taken together, this study revealed that PLR was a significant prognostic factor for poor OS and invasiveness in DLBCL patients. To our knowledge, this is the first meta-analysis investigating the prognostic and clinicopathological value of PLR in DLBCL. Notably, the eight included studies are retrospective study design and recruited patients with one ethnicity. In the present meta-analysis, we collected the data and conducted subgroup analysis to investigate the prognostic value of PLR in different ethnicity, sample size, and cut-off values populations. We also investigated the correlation of PLR and clinical features with enlarged sample size compared with included studies. The current meta-analysis provides more comprehensive and systemic analysis than any single included study. Those factors were strengths of this meta-analysis.

Recent evidence suggests that inflammation response is involved in the processes of tumor angiogenesis, tumor growth, and metastasis [25]. However, the mechanism underlying the correlation between high PLR and poor prognosis in DLBCL patients has not been determined. The increasing of platelet counts and/or decreasing of lymphocyte counts can result in a high PLR. On the one hand, activated platelets were involved in early and advanced stages of tumor angiogenesis [26]. Platelets could secrete various biological molecules to facilitating angiogenesis in tumor microenvironment [27]. In addition, platelets derive transforming growth factor-β1 (TGF-β1), work together with the direct interaction of platelets and tumor cells to activate epithelial–mesenchymal transition (EMT) related pathways and induce subsequent metastasis [28]. On the other hand, lymphocytes exert critical roles in antitumor immune responses. Tumor-infiltrating lymphocytes (TILs) including CD3+ T cells, CD8+ T cells, Th1 CD4+ T cell could inhibit tumor cell proliferation and metastases [29, 30]. Therefore, it is reasonable to apply PLR as an easily available immunological parameter to predict survival outcomes in cancer patients.

Previous studies also demonstrated the prognostic value of PLR in various tumors [31]. A recent meta-analysis showed that a high NLR was significantly associated with decreased OS and PFS in ovarian cancer [9]. Another work suggested that higher PLR was an indicator of poor progress in oral cancer [32]. Those findings were in accordance with our results in DLBCL. In addition, in the present meta-analysis, we found that PLR was a significant prognostic factor for OS, especially in Asian patients and PLR > 150. Those results suggest that PLR may have enhanced prognostic role when the cut-off value > 150, which provides implications for clinical use. An elevated PLR was also correlated to aggressive tumor characteristics, which may imply that DLBCL patients with high PLR should be treated with strong therapeutic strategies. However, we did not observe significant prognostic impact of PLR on PFS, which may be explained by the relative short follow-up of PFS, compared to OS. In addition, the results suggested that cell of origin had non-significant association with PLR. However, because only two studies were included for analysis, which may lead to the negative results, therefore, more large-scale studies are still needed.

There are several limitations to this study. First, significant heterogeneity was observed in the analysis, although we applied random-effect model accordingly. Because the included studies recruited patients with different ethnicity, disease stage, cut-off values and treatment strategies, which could result in heterogeneity in the meta-analysis. The subgroup analysis showed that significant heterogeneity still exists in different sample size and cut-off values groups. These indicated that the heterogeneity could be inherent among included studies and various cut-off values may be a source of heterogeneity. According to the heterogeneity, we selected corresponding effects model (random effects model or fixed effects model) to pool the data. Second, the cut-off values of PLR were different in included studies, which may influence the distribution of low and high PLR groups and cause heterogeneity. Third, we only included studies published in English and Chinese, therefore, relevant studies published in other languages may be unavailable.

## Conclusion

Our study shows that high PLR was a significant prognostic marker for poorer OS in DLBCL. Furthermore, PLR was associated with presentation of B symptoms, elevated LDH, higher tumor stage, and ECOG PS ≥ 2. Considering the above-mentioned limitations, large-scale prospective studies with uniform cut-off value of PLR are needed to validate our findings.

## Data Availability

The data that support the findings of this study are available from the corresponding author upon reasonable request.

## Abbreviations

CI: confidence interval
HR: hazard ratio
NOS: Newcastle–Ottawa Scale
OS: overall survival
PLR: platelet-to-lymphocyte ratio
PFS: progression-free survival
PRISMA: Preferred Reporting Items for Systematic Reviews and Meta-Analyses
DLBCL: diffuse large B-cell lymphoma
R-CHOP: rituximab, cyclophosphamide, doxorubicin, vincristine, and prednisone
LDH: lactate dehydrogenase
ECOG PS: Eastern Cooperative Oncology Group performance status
